# First steps towards semantic descriptions of electronic laboratory notebook records

**DOI:** 10.1186/1758-2946-5-52

**Published:** 2013-12-20

**Authors:** Simon J Coles, Jeremy G Frey, Colin L Bird, Richard J Whitby, Aileen E Day

**Affiliations:** 1 Chemistry, Faculty of Natural and Environmental Sciences, University of Southampton, Southampton SO17 1BJ, UK; 2 Royal Society of Chemistry, Thomas Graham House, Science Park, Milton Road, Cambridge CB4 0WF, UK

## Abstract

In order to exploit the vast body of currently inaccessible chemical information held in Electronic Laboratory Notebooks (ELNs) it is necessary not only to make it available but also to develop protocols for discovery, access and ultimately automatic processing. An aim of the Dial-a-Molecule Grand Challenge Network is to be able to draw on the body of accumulated chemical knowledge in order to predict or optimize the outcome of reactions. Accordingly the Network drew up a working group comprising informaticians, software developers and stakeholders from industry and academia to develop protocols and mechanisms to access and process ELN records. The work presented here constitutes the first stage of this process by proposing a tiered metadata system of knowledge, information and processing where each in turn addresses a) discovery, indexing and citation b) context and access to additional information and c) content access and manipulation. A compact set of metadata terms, called the elnItemManifest, has been derived and caters for the knowledge layer of this model. The elnItemManifest has been encoded as an XML schema and some use cases are presented to demonstrate the potential of this approach.

## Background

The Chemistry Grand Challenge, Dial-a-Molecule [1] is an academic think tank and network with the 20–40 year aim of making the delivery of novel chemical compounds a matter of days as opposed to the years it may currently take. The roadmap [2] for effecting this transition charts a variety of advances needed, but central is the ability to predict the outcome of novel chemical reactions. Despite the vast number of reactions reported over the past century, tens of millions of which are included in electronic databases [3], such prediction is currently very unreliable. A consequence is that in multi-step synthesis many steps will need substantial experimental work to find an acceptable method, and many forced changes to the initially envisaged route before successful completion is the norm. One reason is that the available published data tends to include just the most successful example of a particular transformation. Sub-optimal results are rarely included and there is no culture of publishing the “negative” results, both of which are crucial to successful modeling of reaction scope and robustness. Furthermore, even the reactions which are published lack information on many of the factors which may be important in determining outcomes. With the rapid movement to the use of Electronic Laboratory Notebooks to capture process and outcomes of reactions the information needed to tackle the central challenge of Dial-a-Molecule is increasingly being collected, but discovery of records of interest and the ability to automatically process them, is a substantial challenge. Apart from confidentiality and IP issues, the use of a wide variety of ELNs, each with their own, often proprietary, data structures, is a significant barrier. The importance of being able to mine Electronic Laboratory Notebooks was recognized by the Pistoia Alliance of major pharmaceutical companies [4] which made determining the feasibility of developing a common query method the topic for one of their first working groups.

After examining the factors that currently influence research data management, we consider the role of the Electronic Laboratory Notebook (ELN) and how data preserved in ELNs might be made available in an open format. We extend this analysis to propose a manifest for describing ELN records in a machine-readable form.

Academia is increasingly being held accountable for the management of the research data it generates. Funding bodies now mandate researchers and their institutions to provide infrastructure to support research data management, owing to the requirement to hold such information for some considerable time after projects finish. UK and US policies [5] comprise high level generic frameworks within which there are specific chemistry implementations. This is being received with some trepidation, but the general mood is that some infrastructure will have to be put in place otherwise funding could be potentially put in jeopardy. Additionally there now numerous requirements from funders [6] that these research outputs are made openly available. There are also indications, particularly in the recent Finch report [7] that there should be policy development to support Open Access through both the ‘author pays’ and the ‘institutional repository’ routes. In response the UK government recently announced that all publicly funded research will be made openly available [8] and also the research councils announced a new policy to this effect [9]. The Finch report and a recent Royal Society commissioned report [10] also state that both Open Access publishers and academia should seek to innovate in the area of research data publication.

Making research data (openly) available in well-defined formats has many benefits, some primary ones being:

•Raw data becomes open to scrutiny by other researchers, which helps to uncover cases of scientific fraud or the more innocent, but equally damaging, mistake or incompetent analysis of otherwise valid raw data.

•It makes previously measured data available for further analysis without the necessity to re-measure the same sample.

•Data with similar characteristics or about a common theme can be collected to form a body of information that can be queried in ways that individual data cannot.

•It promotes interdisciplinary research.

•Data can be mined in previously unknown ways or new methods applied to existing data.

Just a few decades ago experimental data was part of the primary publication, however it saw a transition to Supplementary Information as volumes became too large to be incorporated into the body of these articles – to the point that journals now expect full experimental details to be provided through this route. However, research data is rapidly gaining attention again and there are emergent initiatives to move this information from the relative obscurity of ‘Electronic Supplementary Information’ associated with an article to the position of being part of the primary literature in its own right, although not contained in the article itself. For example Lab Archives have developed a relationship with the BioMedCentral publisher [11] to better support the publication of laboratory observations in biological disciplines, the FigShare [12] initiative is born out of a publishing background and the RSC’s ChemSpider [13] and ChemSpider SyntheticPages [14] provide a means to upload and publish structures, spectra, reactions and reaction optimisation know-how. However none of these developments enable the seamless and complete crawling and harvesting of information as it is spread over many different systems, sometimes subject to subscription-based access and never presented in a form that is automatically digestible by machines. Also there is a significant burden on the submitting authors to structure and upload information and the process of making it available would be simpler and scientifically more rigorous if the original laboratory record could be directly used for this purpose.

It has been acknowledged at the highest level [15] that “research data are heterogeneous, often classified and cited with disparate schema, and housed in distributed and autonomous databases and repositories. Standards for descriptive and structural metadata will help establish a common framework for understanding data and data structures to address the heterogeneity of datasets.” This is equally the case with the data held in ELNs.

Electronic Laboratory Notebooks (ELNs) are a common way of gathering raw, derived and observational data and are ubiquitous in many commercial settings, but relatively emergent in academia. There are numerous ELNs currently on the market [16], although the industry developing this software is undergoing a period of change due to the general push for economic efficiency and altering models for the provision of infrastructure and support software. This is causing a review of business models and service delivery for the software providers. One potential new business route is to establish ELNs in academic practice. In general traditional ELNs don’t readily lend themselves to the academic environment as they have been built to suit commercial purposes. The drivers for academic use, most notably the motivation to publish work, are somewhat different to those of the commercial setting, which is primarily concerned with the protection of IP. However it is clear that many of the compelling reasons for adopting an ELN are very appropriate in an academic setting. However, it should be pointed out that there is room for some convergence and overlap between these two ELN environments for example a) in areas of academic research where IP protection is a requirement or b) in industry, particularly Processing, where there are efficiency gains that can be made through sharing data.

Business and policy aside, there are compelling academic research focused reasons to embrace the use of ELNs. To an extent most ELNs will provide a structured recording of research observations in digital form, which encourages scientific literacy, good laboratory practice and safe operating, long term preservation of data and protection of IP. However academia is concerned with progressing understanding by knowledge sharing and ELNs are not well suited to this, but if approached correctly they have the potential to support this process very well.

The degree of accuracy of reporting observations and method in such a way that claims can be reproduced by others is fundamental to scientific integrity. This has been realized through the use of ELNs in industry. An example is alluded to in the description of the implementation of an ELN at AstraZeneca [17] – whilst this refers mainly to efficiency gains, reference is made to cloning experiments for reproducibility and a communication from this company indicated that 85% of ELN records were generated from cloning ones own and that 10% of these clones were shared. This point has also been made many times in the ELN developer community when comparing paper notebooks to ELNs and is perhaps best summarized by Michael Elliot’s 2005 webinar [18].

Without such high levels of accuracy being employed it is difficult for work to form the basis for further research and accurate peer review. Current practice in supporting conclusions drawn from experimentation is to provide the associated data gathered, however this informs little about the method. It is relatively common for there to be inconsistencies with claims made in submitted journal articles, poorly conducted experiments or flawed reasoning and publishers are becoming aware of this problem and are beginning to address it [19]. The so-called “ClimateGate” affair [20], where scientists allegedly manipulated climate data to silence critics, could have been significantly less serious if the academics concerned had revealed more about the methodology they employed and thus demonstrated that it was in accordance with community practice. In some cases academic fraud has been uncovered due to publishers requiring full deposition of data related to submitted articles, which has enabled rigorous procedures to analyse the integrity [21] of that data [22,23].

The Dial-a-Molecule network has set up a working group to address this issue, which proposes a high-level semantic description of an ELN record that enables discovery and dissemination and assessment of accessibility and automated processability. This paper promotes the notion of the ELN as a ‘publishing’ platform in its own right. Operating as such, ELN records could either be made available independently or be linked to and from formal or traditional publications. This would mean that in principle all laboratory observation could be made available in a complete and original form for the greater academic good. For this approach to scale in a sensible fashion for automated harvesting and linking there is the need for protocols to describe the content in a concise and standardised form so that consumers can easily and automatically decide if the information being made available is of relevance to them or not. Moreover, there is becoming a recognised need for better exporting of data from ELN’s. This is not only so that data can be mined or transported between different platforms for sharing, but also the presentation and structured availability of ELN records as part of the publishing process and scientific record. This is illustrated by the following quote in a recent review article which is an observation based on an earlier comment by Macneil [24].

“However, five years later, concerns remain about difficulties with exporting data from most ELNs. Overall, this post addresses the question of handling the research data associated with publications, very much a topical issue, but one not frequently raised in the context of ELNs”.

The work presented herein describes a machine-readable mechanism for enabling these processes that is tailored particularly for the data held in ELNs and provides two working implementations exemplifying its use.

### Previous work and inspiration

The institutional repository (IR) development community provides a close relation to the ELN in that these resources contain the outputs of research and are designed to report their content in order for citation and linking services to discover relevant records. Moreover these resources also provide a preservation function, much like the requirement for an ELN, and are generally developed by the library and information sciences community whose role is to store, catalogue and retrieve records. This community has coherently developed a records description standard that has been in common in practice for some years – Dublin Core, DC [25]. DC itself comprises a set of 15 metadata elements that describe the records held in an IR. It should be stressed at this point that these systems and resources are generally built to contain the outputs of research (predominantly journal articles) and their primary purpose is to provide a dissemination mechanism. Increasingly IRs are being used to preserve the institution’s research outputs and establish a degree of ownership over them. As such these descriptions serve as a mechanism to advertise the content and conform to a harvesting protocol, OAI-PMH [26], that allows agents acting for centralised search and discovery services to find records of interest and gather metadata or potentially even the data itself. The records in these repositories can be described by DC through a second tier known as Qualified DC – this extension enables a repository to develop an extra set of metadata that permits more accurate and detailed descriptions of less conventional content. Qualified DC has been shown to be adaptable to describe very particular scientific information eg the eCrystals Repository [27]. There are other similar approaches that make use of metadata application profiles such as the Dryad Data repository [28] which provides a generic platform to independently make data available – however it is generally data associated with published journal articles.

DC is therefore lightweight by design, however Learning Object Repositories are a closer reflection of ELNs in that they hold complex digital objects as opposed to simple, well understood, documents. These objects are more akin to the records held in ELNs in that they generally contain several discrete (editable) components: content, learning activities and context [29]. Being more complex, these objects have more specific and detailed metadata for the purposes of discovery, reusability and interoperability such as educational objective, prerequisites, topic, interactivity and technology requirements. The IEEE have produced a standard [30] in order to ensure uniformity across all Learning Object resources.

Simply making data available without being explicit about terms of use can cause problems – not least as the default legal position is rarely obvious and can depend heavily on jurisdiction. An ELN can be likened to a database in that it may comprise a schema, fields, tables and the data itself and all of these may be used by different parties in a number of different ways. This phenomenon is known to create considerable complication in respect of copyright law and protection of Intellectual Property. For example, within the European Union a database creator employs intellect, skill and expert judgment in its creation and therefore the database itself is subject to copyright [31]. ELN data made openly available should therefore carry with it a license that unequivocally details how they may be used. It is important to note that an ELN record often consists of multiple components that have been drawn from various different departments, disciplines, creators, etc. and in many cases it will be far from clear as to who the rights holders are. In certain circumstances it may be possible or sensible for an author to explicitly waive all rights to the data, by means of a waiver. Otherwise, a bespoke license may be generated or a standard one can be employed. Generating a bespoke license is by no means a trivial task and it is most likely that the only pragmatic and scalable solution will be to use a standard one. It is beyond the scope of this article to detail properties and suitability of the numerous standard licenses that may be applicable to ELN data, however, a comprehensive practical guide has been generated by the Digital Curation Centre [32].

The citation of data is currently a topic of intense interest and debate now that more data is being made available and hence the ability to link and acknowledge is necessary. Not only is “Adequate citation of data sets crucial to the encouragement of data sharing, to the integrity and cost-effectiveness of science and to easy access to the work of others” [33], but also “Without an effective data citation mechanism the implementation of the ‘Data Publishing Framework’ would remain incomplete. Thus, universal standards for citing datasets are essential [34]”. A concise background to this topic is provided by Ball and Duke [35]. The metadata scheme provided by DataCite [36] is emerging as a standard in this area and reflects very closely a subset of DC. By design data citation formats must be concise and therefore contain only a small number of descriptive data, but in the design of a scheme for ELN data it is important to be compliant with this approach.

The approaches outlined above do not contain information about process or provide a detailed description of the actual data itself. For many applications, most notably the automated processing necessary to enable Dial a Molecule, this is crucial. A pertinent example of this extra level of detail and thereby the ability to write more complicated software to process the information being made available is the Core Scientific Metadata Model (CSMD) [37] and related implementations that have been developed by STFC to support the experimentation being undertaken at large central facilities and laboratories. This work is particularly embodied in the PaN-data initiative [38] where centralised facilities across Europe have agreed to a common policy framework [39] which also enables common terms to be defined and agreed.

The most relevant prior work, which is represented through membership of the Dial a Molecule working group, is that of the Pistoia Alliance [4]. One of the Pistoia Alliance’s first endeavours was to determine the feasibility of developing an ELN query service for accessing different vendor ELNs. This work developed use cases and engineered an initial prototype of the service, and the learnings from this group are informing the development of a broader chemistry strategy. Envisioned outcomes include the development of a universal chemistry query, ELN datamarts, and hosted services for managing the chemistry supply chain and in vitro and in vivo screening data. This model is built on a very comprehensive relational and hierarchical vocabulary. However, this vocabulary is built entirely on the requirements of the pharmaceutical industry as served by the current ELN vendors and as such does not cover significant areas that would be necessary for academic use.

In essence an ELN for academia should:

•generically support a range of disciplines

•develop a data management framework

•support a range of data acquisition techniques at different scales (complexity, volume, definition)

•promote common and easy access to data, sharing and reuse

•enable discovery of results in related disciplines

•facilitate access to data underpinning publications, resulting in higher levels of verification & quality

•enhance rapid communication across the community

•support long-term preservation, assuring future discovery of results and needless reproduction of work

To comprehensively meet the challenges posed by the Dial a Molecule network there are some specific requirements of a metadata scheme. The working group reviewed the Dublin Core scheme and elected to devise a bespoke system for the following reasons.

•The corpus of data containing information about reactions and their conditions is spread across academia and industry and held in a number of very different resources, such as patent databases, laboratory notebooks, reports and journal articles. A data description must be applicable to all these and particularly when considering access regulation it was deemed important to have a contact, and that this is not necessarily the author.

•Paper laboratory notebooks will still be in operation for some time to come in certain areas and a scheme must be able to refer to, and subsequently process these physical entities in some way. It is therefore important to accurately identify the source of the information (and potentially contacts etc. in order to process it further).

•Each record must be able to have numerous date types associated with it in order to protect IP, eg when an idea was conceived, the work conducted, the work submitted to an ELN, when an embargo can be lifted, when is metadata available, when a record may be automatically mined and processed.

•Laboratory notebook records are highly specific to a (sub)discipline, heterogeneous, complex in nature and often in proprietary formats. A system must be able to indicate the nature of the unit - i.e. multiple ELN records, an individual record, or a component of record, such as a spectrum or reaction. It must also be able to package and describe the unit.

•Laboratory notebooks are fundamentally concerned with recording the process undertaken - be it by a researcher, an instrument, or by software. This information needs to be appropriately captured, described and made available for the Dial a Molecule Grand Challenge to be a success.

•Records must be described, structured and formatted in order to enable their automated discovery and processing – it is only through automation that the Dial a Molecule vision can be realised.

The work presented in this paper outlines a high-level descriptive metadata scheme. This simply enables discovery – the follow-on access and processing steps require a much more detailed and discipline specific scheme. This requirement does however have implications on the design of the discovery scheme, particularly in respect of packaging and providing access to subsequent tiers, and therefore a bespoke solution was chosen. Moreover, previous experience of applying generic metadata schemes to specific scientific data sets, such as the eBank project [40], highlighted the limitations of Dublin Core. The main barrier is that a general scheme can only be applied to datasets that are well understood in advance and highly structured and that it is not trivial to capture process information [41].

## The approach

The work outlined in the section above presents numerous ways of describing records in data management systems concerned with academia and chemistry – from very brief to very comprehensive. However no single one of these approaches is entirely suited to all the requirements for a set of descriptive data about an ELN record – e.g. DC is too generic and minimal for automatic data processing, whilst at the other end of the spectrum the Pistoia Alliance work has gaps and lacks the ability to support lightweight “transactional” processes.

The IDMB project [42] was concerned with a university institutional view to data management and devised a tiered approach to metadata architecture, based on the following levels:

1) ‘core’ metadata for discoverability, akin to the 15 Dublin Core elements, used for DOI registration, etc. This could be considered a generic *knowledge layer* and would answer questions such as “What is being made available and is it of interest to me?” and “Can I access it?”.

2) ‘contextual’ metadata which essentially covers the elements in CERIF, [43] notably a) outcomes i.e. publications, patents; b) funding e.g. research council grant c) people e.g. project team members, d) organisation i.e. University, collaborators. This could be considered as the *information layer* and would answer questions such as “At what granularity should data be made available or citable?”, “What is an ELN record?” and “If single datasets are given identifiers, what about collections of datasets, files within datasets or individual data?”.

3) ‘detail’ metadata - a specific level giving the minutiae such as formats etc. and that would enabling (potentially automatic) processing. This could be considered as the *processing layer* and would answer questions such as “Can I automatically process this information?”.

This approach has been adopted by a number of high profile projects. This three layer metadata model is also being used in some ESFRI [44] projects such as EPOS and ENGAGE. In this scheme the knowledge layer is for casual browsing in a Linked Open Data environment using a combination of Dublin Core, CKAN [45] and eGMS [46]. This layer is generated from the middleinformation layer which uses CERIF in a conventional research information system environment, including datasets but linked to persons, organisations, projects, funding, facilities, equipment etc. The CERIF, in turn, points to detailed metadata in the processing layer for particular domains of research e.g. CSMD for data from Neutron and Synchrotron sources [47].

### Implementing a system for ELNs

In the context of ELNs it is appropriate to consider the layered approach, due to the scale and complexity of the information they contain and the various different ways in which it may be accessed or reused. To the best of our knowledge at the time of writing there is no immediately applicable model or system that could be generically applied to all types of ELN irrespective of vendor or discipline. The following section therefore presents a model for implementing the three-layered system for ELNs. It comprises a specifically devised knowledge layer, the elnItemManifest, and indicates ways in which it permits access to underlying information and processing layers.

The description presented in this paper, named the *elnItemManifest*, comprises a compact set of terms including title, keywords, identifiers, contact, license information, related items, contributors, content, source and dates. These terms comprise the core metadata and thus represent the knowledge layer of the model described above. We present a validated XML schema that represents this information – the full schema is provided as supplementary information and made available via http://www.dial-a-molecule.org/wp/blog/2013/08/elnitemmanifest-a-metadata-schema-for-accessing-and-processing-eln-records/^a^. The schema has been designed to be pragmatic and tractable for current practice (as discussed by the Dial-a-Molecule network). Accordingly there are a number of elements in the schema that are not machine readable at this point in time – this is in part to allow a greater degree of flexibility at a stage when the whole community is not yet ready for this degree of automation and in part because this requires an *agreed* controlled vocabulary and it is premature for this consensus to be reached.

The fields, which are mandatory unless specified as optional, with a brief description, are described in Table 1.

**Table 1:** The content of the elnItemManifest

**Element**	**Description**	**Qualifier**	**Notes**
elnItemManifest	Definition of the ELN item manifest itself	UnitType	Allow a system to indicate the nature of the unit - i.e. multiple ELN records, an individual record, or a component of record, such as a spectrum or reaction.
		Package	
		Record	
		Component	
Title	Short, human-readable, text string to assist when viewing this record in a list: helps the reader to determine whether record is of interest		
Keywords	[Optional] Text strings that might assist in searching or categorising	KeywordSet	A list of terms
Identifiers	Unique handles that identify this record	IdentifierSet	Primary string, URI, or item in any other format that enables this record to be located uniquely in the originating system
		PrimaryLocalIdentifier	
		OtherLocalIdentifier	[Optional] Alternative means of locating record in the originating system
		AccessIdentifier	[Optional] URI that provides a direct link to the content. If included, must be a ‘linked data’ URI giving open access
Contact	Specifies who or what to contact for more information.	ContactOption	Could be: e-mail address, system URI, or brief instruction. Expect to get some sensible reply when contacting this person or system with the localIdentifier specified.
		EMail	
		SystemURI	
		Instruction	
LicensingBasis	[Optional] Indication of basis for licensing		e.g. Creative Commons
Contributors	List of contributory people, organisations, etc.	ContributorSet	For example, Author, Funding Body, PI, institution. Plain text, but name ideally complemented by unique identifiers
		ContributorInformation	
		Role	
		Name	
Source	String describing the system that generated this data.		Generally identifies vendor, software package and version (resembles a browser user agent)
Date	One or more datestamps	DateSet	Dates on which this record was created; on which any embargo ends; for associated publication; or submission to conference, journal etc.
		CreationDate	
		ReleaseDate	
		PublicationDate	
		SubmissionDate	
RelatedItems	[Optional] List of items that might be related	RelatedItemSet	Nature of the related information, for example, publication or related work
		Relationship	ID can be any string, but DOI preferred if the related item is a publication. Zero or more item(s) of related information
		Id	
Content	Items comprising the record that this describes	ContentInformation	A list that may comprise descriptions, file types and links to data

The elnItemManifest is structured so as to be compliant with the three-tier model described above.

•The manifest itself is intended to take the role of the *Knowledge Layer* – this layer is intended to be a summary of the content of a record and would enable any person or agent to rapidly assess whether a record is of interest. Indexing and cataloging services eg for data citation or discovery would be able to directly use this information without further processing, whereas agents wishing to mine the data itself or incorporate it into a database or collection would be able to immediately assess whether the record is of interest and accordingly whether to continue to the information or processing layer in order to do so. The manifest itself is generic and therefore independent of the specific format of the content or the type of ELN and can span or transcend disciplines. The elnItemManifest could be harvested in a similar fashion to OAI-PMH, packaged similarly to OAI-ORE and make use of approaches like SWORD for automatic deposition.

•The *Information Layer* is seen to be performing the role of providing additional context or access to additional or related information. In the elnItemManifest the ‘relatedItems’ element is seen as the route into this layer and it is expected that this is utilised by administrative systems eg as a catalogue that can connect data with experiments, facilities, funding, publications, etc. or by aggregator services that, for example, connect data and publications.

•The ‘content’ element in the elnItemManifest is essentially the access point to the *Processing Layer* and it is expected that agents wishing to automatically obtain or process the data itself would use this. It is envisaged that the ‘content’ is a list that may be comprised of a) a description such as a text field which is human readable and describes what the item is, b) the data type e.g. a MIME type which says what this digital object is.

In addition to the two content components, a dereferencable link to the data itself may be optionally included (via the accessIdentifier element, which is a constitutent of identifierSet). Through this mechanism, based on the fact that it is interested in a particular MIME type file, an agent could potentially follow the link to the data and automatically process it. To enable such automation, the content element could direct to a very semantically defined and structured file type eg CML(and its extensions) [48], CIF [49], AniML [50] or to an ontology eg RXNO, CMO, MOP [51], ChEBI [52] or a structured vocabulary such as CSMD [33].

### Scenarios and demonstrators

In order to provide some context for how the elnItemManifest could work we present some scenarios of use – we consider these to be examples of primary uses of this approach. We also provide elnItemManifest examples and some rudimentary demonstrators associated with two different ELN’s – the LabTrove [53] and IDBS [54] systems.

#### Scenario 1 – Publication and dissemination

The first scenario is one where the ELN advertises its content i.e. it acts as a publishing platform in its own right and this can be envisaged as having two modes of operation

a) The ELN can provide direct support to the conventional publishing process ie by providing access to supplementary information. In this case the metadata relating to a record is harvested by the publisher from the ELN and used as a pointer to the data from the article.

b) Alternatively the ELN can act independently and broadcast information about the records it contains through the elnItemManifest mechanism. An example of using this approach might be where a second party, such as a centralized service that links data and publications or aggregates datasets, finds or subscribes to the elnItemManifest feed and requests access to the record. The data contained within the ELN record is accessed by the service provider, deemed as appropriate and then the metadata harvested and held as a pointer to the original record in the ELN.

#### Demonstrator 1

The LabTrove ELN has the capacity to act as a publication platform – this ELN can openly publish records and correspondingly make this information available according to the elnItemManifest schema, thereby providing a structure that can be readily be processed by information gathering ‘robots’. Additional file 1 provides an elnItemManifest that corresponds to the LabTrove record at the following address http://www.ourexperiment.org/synth_methyl_oxin/5606/Spectrum_of_31Bromophenyl_1_3Dihydroindol2Hone.html.

#### Scenario 2 – Seamless contribution or submission to a database

The second scenario provides an example of automated (initial) deposition into a centralised repository, containing a description of the ELN item and a link back to its source. After discovering the existence of a record, by either ‘push’ or ‘pull’ mechanisms involving elnItemManifests, i) a database, such as ChemSpider, *could* ingest data of interest and automatically incorporate it wholly into its database or ii) an agent *could* programmatically extract excerpts of the dataset it requires. The latter would be an example of how the Dial-a-Molecule vision could be put into practice.

#### Demonstrator 2

A plug-in has been written for the IDBS e-Workbook, which generates the elnItemManifest and makes it available for further use.

A previous plugin had been written for IDBS’s e-WorkBook ELN, which allowed chemical structures which are part of an ELN experiment to be published to ChemSpider. The initial version of the plugin sent a single SDF file per deposition which contained both the chemical structures (in mol format) and some very basic metadata information about where it comes from (author, principal investigator, ELN experiment ID) in the associated data fields. For this demonstrator the plug-in was adapted to also generate the metadata as a separate elnItemManifest file (to be viewed and saved). Additional file 2 shows the elnItemManifest as generated by the plugin for an example ELN record stored in the IDBS installation for the Chemistry department at Cambridge University (who kindly allowed this plugin to be developed against their ELN installation). Where possible the fields in this elnItemManifest were extracted programmatically from the ELN system. Exceptions to this are the contact and contributors’ details which are saved in a configuration file for the plugin (that can be edited and saved via the ELN interface) and the licensingBasis and embargo period (from which the releaseDate was calculated) which are input by the user when the elnItemManifest is generated (since these may change from one deposition to the next). More details about this plugin and how to obtain it are detailed on the ChemSpider blog at http://www.chemspider.com/blog/chemspider-eln-plugin-generates-elnitemmanifest.html and a video demonstration of it can be viewed at http://youtu.be/MwyecFHRolI.

It should be noted that this plugin was simply a first step to demonstrate the generation of the elnItemManifest from an ELN, and it is not currently sent to ChemSpider to accompany a deposition to automatically mark the provenance of those deposited structures. The next step would be to develop the ChemSpider deposition service so that this was possible. The driver for this development for ChemSpider would be that it would make it much easier to integrate other ELNs to be able to deposit to ChemSpider, rather than writing a separate plugin for each, particularly if the vendors and developers of other ELNs built the ability to generate this metadata into their APIs. Separating out the metadata from the data file itself would also make it simpler to use the same deposition system for all sorts of data from ELNs, such as spectra, reactions and properties.

## Conclusions

ELNs support a vast variety of disciplines, information and data types and exist on many different software platforms and systems. Moreover they exist in a complicated and varied information exchange environment, which is in emergent stages of development for academia. It is therefore timely and important to devise approaches to managing, making available and processing this information. Herein we present a model and approach that can be generically implemented.

The elnItemManifest is a high level metadata description for ELN information that can be used for discovery, exchange and reuse purposes and addresses the highest, knowledge, layer in the model. The next stages in the development of this model, which we have shown the elnItemManifest can access and have described some aspects of, are to develop metadata support for the information layer such that it will become possible for the processing layer to be accessed.

The elnItemManifest is published as a versioned XML schema definition and may be accessed via the Dial-a-Molecule website (http://www.dial-a-molecule.org/wp/blog/2013/08/elnitemmanifest-a-metadata-schema-for-accessing-and-processing-eln-records/) and can be implemented in any ELN system.

## Endnote

^a^In order to provide assured version control of the schema and associated examples, it is currently hosted on a local code trunk of the LabTrove software, but accessibility is advertised via the Dial-a-Molecule website.

## Competing interests

The authors declare that they have no competing interests.

## Authors’ contribution

Following a Dial-a-Molecule working group recommendation CB, assisted by SC and advised by all authors, drew up the XML scheme and LabTrove exemplar. AD developed the IDBS exemplar. SC drafted the manuscript and all authors made significant comment and contribution. All authors read and approved the final manuscript.

## Supplementary Material

Additional file 1 An elnItemManifest corresponding to a LabTrove record. Click here for file

Additional file 2 An elnItemManifest generated by the plugin for an example record stored in the IDBS ELN. Click here for file
