# Supplementary material for: First steps towards semantic descriptions of electronic laboratory notebook records
Source: J Cheminform. 2013 Dec 20;5:52. doi: 10.1186/1758-2946-5-52 (PMC3878183; doi:10.1186/1758-2946-5-52)
Supplement: Additional file 1 — An elnItemManifest corresponding to a LabTrove record. [file 1758-2946-5-52-S1.docx]

**Appendix 1.**

LabTrove elnItemManifest produced for record <http://www.ourexperiment.org/synth_methyl_oxin/5606/Spectrum_of_31Bromophenyl_1_3Dihydroindol2Hone.html>

<?xml version="1.0" encoding="UTF-8"?>

<elnItemManifest schemaVersion="1.0" unit="package" xmlns:xsi="http://www.w3.org/2001/XMLSchema-instance" xsi:noNamespaceSchemaLocation="elnItemManifest.xsd">

<title>title</title>

<keywords>

<keyword>Spectroscopic Method</keyword>

<keyword>Substituent</keyword>

</keywords>

<identifiers>

<primaryLocalIdentifier>5606</primaryLocalIdentifier>

<otherLocalIdentifier>Optional: unable to devise one</otherLocalIdentifier>

<accessIdentifier>http://www.ourexperiment.org/synth_methyl_oxin/5606/Spectrum_of_31Bromophenyl_1_3Dihydroindol2Hone.html</accessIdentifier>

</identifiers>

<contact>

<eMail>S.J.Coles@soton.ac.uk</eMail>

</contact>

<licensingBasis>Own Work: Released into the public domain (under Creative Commons CC0 license)</licensingBasis>

<relatedItems>

<item>

<relationship>Image file of mass spectrum</relationship>

<id>http://www.ourexperiment.org/data/files/4181/MS%20bromo.png</id>

</item>

</relatedItems>

<contributors>

<contributor>

<role>Author</role>

<name>Nicola Knight</name>

</contributor>

</contributors>

<content>

<description>Spectrum of 3-(1-Bromophenyl) 1, 3-Dihydroindol-2H-one</description>

<mimeType>text/plain</mimeType>

</content>

<source>LabTrove 2.3-r455 (Aurora)</source>

<dates>

<creationDate>2012-05-06</creationDate>

<releaseDate>2012-05-06</releaseDate>

<publicationDate>2012-05-06</publicationDate>

</dates>

</elnItemManifest>
