# Supplementary material for: First steps towards semantic descriptions of electronic laboratory notebook records
Source: J Cheminform. 2013 Dec 20;5:52. doi: 10.1186/1758-2946-5-52 (PMC3878183; doi:10.1186/1758-2946-5-52)
Supplement: Additional file 2 — An elnItemManifest generated by the plugin for an example record stored in the IDBS ELN. [file 1758-2946-5-52-S2.docx]

**Appendix 2.**

ChemSpider elnItemManifest produced for example IDBS experiment (see <http://www.chemspider.com/blog/chemspider-eln-plugin-generates-elnitemmanifest.html> for more details)

<?xml version="1.0" encoding="UTF-8" standalone="no"?>

<elnItemManifest xmlns:xs="http://www.w3.org/2001/XMLSchema">

<title>Test01 - Diels-Alder reaction</title>

<keywords>

<keyword>cycloaddition</keyword>

<keyword>conjugated diene</keyword>

<keyword>substituted alkene</keyword>

<keyword>substituted cyclohexene</keyword>

</keywords>

<identifiers>

<primaryLocalIdentifier>aeg20.110826</primaryLocalIdentifier>

<otherLocalIdentifier>aeg20.110826</otherLocalIdentifier>

</identifiers>

<contact>

<eMail>daya@rsc.org</eMail>

</contact>

<licensingBasis>Own Work: Released into the public domain (under Creative Commons CC0 license)</licensingBasis>

<relatedItems/>

<contributors>

<contributor>

<role>Author</role>

<name>Aileen Day</name>

</contributor>

<contributor>

<role>Principal investigator</role>

<name>Tim Dickens</name>

</contributor>

</contributors>

<content>

<description>compound mol(s) from 'An example reaction for testing export to ChemSpider'</description>

<mimeType>mol</mimeType>

</content>

<source>IDBS E-WorkBook Suite</source>

<dates>

<creationDate>2011-08-26</creationDate>

<releaseDate>2012-06-16</releaseDate>

<submissionDate>2012-06-16</submissionDate>

</dates>

</elnItemManifest>
